# Chromothripsis is a novel biomarker for prognosis and differentiation diagnosis of pancreatic neuroendocrine neoplasms

**DOI:** 10.1002/mco2.623

**Published:** 2024-07-10

**Authors:** Ming‐Yi Zhang, Du He, Yi Zhang, Ke Cheng, Hong‐Shuai Li, Yu‐Wen Zhou, Qiong‐Xian Long, Rui‐Zhi Liu, Ji‐Yan Liu

**Affiliations:** ^1^ Department of Biotherapy, West China Hospital Sichuan University Chengdu Sichuan China; ^2^ Department of Pathology, West China Hospital Sichuan University Chengdu Sichuan China; ^3^ Center of Life Sciences Peking University Beijing China; ^4^ Department of Pathology, Nan Chong Central Hospital the Second Affiliated Hospital of North Sichuan Medical College Nanchong Sichuan China; ^5^ School of Medical and Life Sciences Chengdu University of Traditional Chinese Medicine Chengdu Sichuan China; ^6^ Sichuan Clinical Research Center of Biotherapy Chengdu Sichuan China; ^7^ Department of Oncology The First People's Hospital of Ziyang Ziyang Sichuan China

**Keywords:** chromothripsis, DDR gene, differentiation diagnosis, pancreatic neuroendocrine neoplasms, prognosis, TP53

## Abstract

This study aimed to identify the role of chromothripsis as a novel biomarker in the prognosis and differentiation diagnosis of pancreatic neuroendocrine neoplasms (pNENs). We conducted next‐generation gene sequencing in a cohort of 30 patients with high‐grade (G3) pNENs. As a reference, a similar analysis was also performed on 25 patients with low‐grade (G1/G2) pancreatic neuroendocrine tumors (pNETs). Chromothripsis and its relationship with clinicopathological features and prognosis were investigated. The results showed that DNA damage response and repair gene alteration and *TP53* mutation were found in 29 and 11 patients, respectively. A total of 14 out of 55 patients had chromothripsis involving different chromosomes. Chromothripsis had a close relationship with *TP53* alteration and higher grade. In the entire cohort, chromothripsis was associated with a higher risk of distant metastasis; both chromothripsis and metastasis (ENETS Stage IV) suggested a significantly shorter overall survival (OS). Importantly, in the high‐grade pNENs group, chromothripsis was the only independent prognostic indicator significantly associated with a shorter OS, other than *TP53* alteration or pathological pancreatic neuroendocrine carcinomas (pNECs) diagnosis. Chromothripsis can guide worse prognosis in pNENs, and help differentiate pNECs from high‐grade (G3) pNETs.

## Graphical abstract

A theoretical model of pNENs tumor progression. DDR alteration occurred at an early stage in pNENs. Then, TP53 deficiency may facilitate cell survival following DNA damage and predispose cells to chromothripsis. Finally, chromothripsis can offer a shortcut to rapid progression.

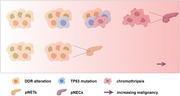

## 1 INTRODUCTION

Pancreatic neuroendocrine neoplasms (pNENs) are a group of tumors characterized by neural antigens. A few of these with mitotic rate > 20/10 HPF or Ki‐67 index > 20% are defined as high‐grade (G3) pNENs.^1^, ^2^, ^3^ To date, some essential issues remain unresolved for these rare but heterogeneous diseases, such as how to identify pancreatic neuroendocrine carcinomas (pNECs) from high‐grade (G3) pancreatic neuroendocrine tumors (pNETs) effectively.^4^ It has been discussed fiercely since several histologically well‐differentiated pNENs with a Ki‐67 index greater than 20% were qualified as pNECs in the World Health Organization (WHO) 2010 classification.^5^, ^6^, ^7^ They still retain many of the features of conventional pNETs.^8^, ^9^, ^10^, ^11^ Therefore, the newest WHO 2017 classification introduced a “pNETs G3” category and distinguished them from poorly differentiated pNECs.^12^, ^13^ This distinction undoubtedly strongly impacts treatment strategies and clinical prognosis, but accurate diagnosis can be challenging due to relying only on a subjective judgment of morphology. For instance, 33 G3 pNENs were examined by three experienced pathologists, but 20 cases (61%) were unable to get a consistent diagnosis.^14^

Other indicators have been proposed to facilitate differential diagnosis, but there are still many flaws and deficiencies. The use of a specific Ki‐67 index threshold (i.e., 55%) to distinguish these entities remains controversial.^15^ The mutations in *TP53* or *RB1* genes were considered characteristics of pNECs, but then extensive sample size sequencing data revealed that *TP53* mutation could be shared by pNECs and high‐grade pNETs.^16^, ^17^, ^18^

On the other hand, carcinogenesis is a process of specific changes to genes that control essential functions of our cells, especially in growing and division.^19^, ^20^ In recent years, with the development of next‐generation DNA sequencing and analysis, new mutational phenomena such as chromothripsis have been identified in cancer cells.^21^ Chromothripsis is a mutational process by which massive genomic rearrangements occur in a single catastrophic event.^22^, ^23^, ^24^, ^25^, ^26^ In contrast to the multistep model for carcinogenesis that cancer is the gradual acquisition of genomic rearrangements and somatic mutations over time, chromothripsis provides a mechanism for the rapid accrual of hundreds of rearrangements in a few cell divisions.^27^, ^28^, ^29^ Thus, it has been associated with highly aggressive tumor behavior except for one case in which chromothripsis spontaneously cured a patient with WHIM syndrome.^30^, ^31^, ^32^, ^33^

This study conducted next‐generation gene sequencing in a cohort of 30 patients with G3 pNENs. As a reference, 25 samples from patients with pNETs G1/G2 were also performed in the sequencing analysis. We aimed to aid in discriminating challenging cases in high‐grade pNENs by examining some new forms of genome alteration, such as chromothripsis.

## 2 RESULTS

### 2.1 Patient clinical and genetic characteristics

A total of 30 patients with high‐grade pNENs were included in our study after histologic review (25 specimens were taken from the pancreas and 11 samples from liver metastasis). According to the WHO 2017 classification for pNENs, the well‐differentiated G3 pNENs were classified as pNETs G3 (*n* = 13), while the poorly differentiated were pNECs (*n* = 13). Four cases were diagnosed as “ambiguous” due to the difficulty of distinguishing between pNETs G3 and pNECs. The representative histopathological images along with corresponding Ki‐67 immunohistochemistry results for these cases are illustrated in Figure S1. To better distinguish between high‐grade (G3) pNETs and pNECs, 15 patients with pNETs G1 and 10 patients with pNETs G2 were also enrolled. As a cohort of 55 patients, the median age was 53 years (range 21−75 years). The proportion of male patients (*n* = 29, 53%) was similar to females. Approximately 73% of patients had symptoms, and 31% already had liver metastasis (ENETS Stage IV) at diagnosis (Tables 1 and S1).

**TABLE 1:** Clinicopathologic characteristics of all patients.

		Low‐grade pNENs		High‐grade (G3) pNENs		
	Total	pNET G1	pNET G2	pNET G3	pNEC	Ambiguous
	(*N* = 55)	(*N* = 15)	(*N* = 10)	(*N* = 13)	(*N* = 13)	(*N* = 4)
	No. (%)	No. (%)	No. (%)	No. (%)	No. (%)	No. (%)
Age, y						
Median	53	54	48	56	53	30
Range	21−75	27−75	26−67	22−71	37−73	21−65
Sex						
Female	26 (47%)	10 (67%)	5 (50%)	6 (46%)	3 (23%)	2 (50%)
Male	29 (53%)	5 (33%)	5 (50%)	7 (54%)	10 (77%)	2 (50%)
Symptoms						
Yes	40 (73%)	8 (53%)	6 (60%)	11 (85%)	12 (92%)	3 (75%)
No	15 (27%)	7 (47%)	4 (40%)	2 (15%)	1 (8%)	1 (25%)
Location						
Head	30 (55%)	7 (47%)	6 (60%)	6 (46%)	10 (77%)	1 (25%)
Body and tail	25 (45%)	8 (53%)	4 (40%)	7 (54%)	3 (23%)	3 (75%)
ENETS stage^a^						
I	5 (9%)	4 (27%)	1 (10%)	0 (0%)	0 (0%)	0 (0%)
II	20 (36%)	8 (53%)	6 (60%)	4 (31%)	2 (15%)	0 (0%)
III	13 (24%)	3 (20%)	0 (0%)	6 (46%)	3 (23%)	1 (25%)
IV	17 (31%)	0 (0%)	3 (30%)	3 (23%)	8 (62%)	3 (75%)
Chromothripsis						
Yes	14 (25%)	1 (7%)	0 (0%)	2 (15%)	10 (77%)	1 (25%)
No	41 (75%)	14 (93%)	10 (100%)	11 (85%)	3 (23%)	3 (75%)

Abbreviation: ENETS, The European Neuroendocrine Tumor Society.

^a^ It was evaluated on the day of pathological diagnosis.

**FIGURE 1:**
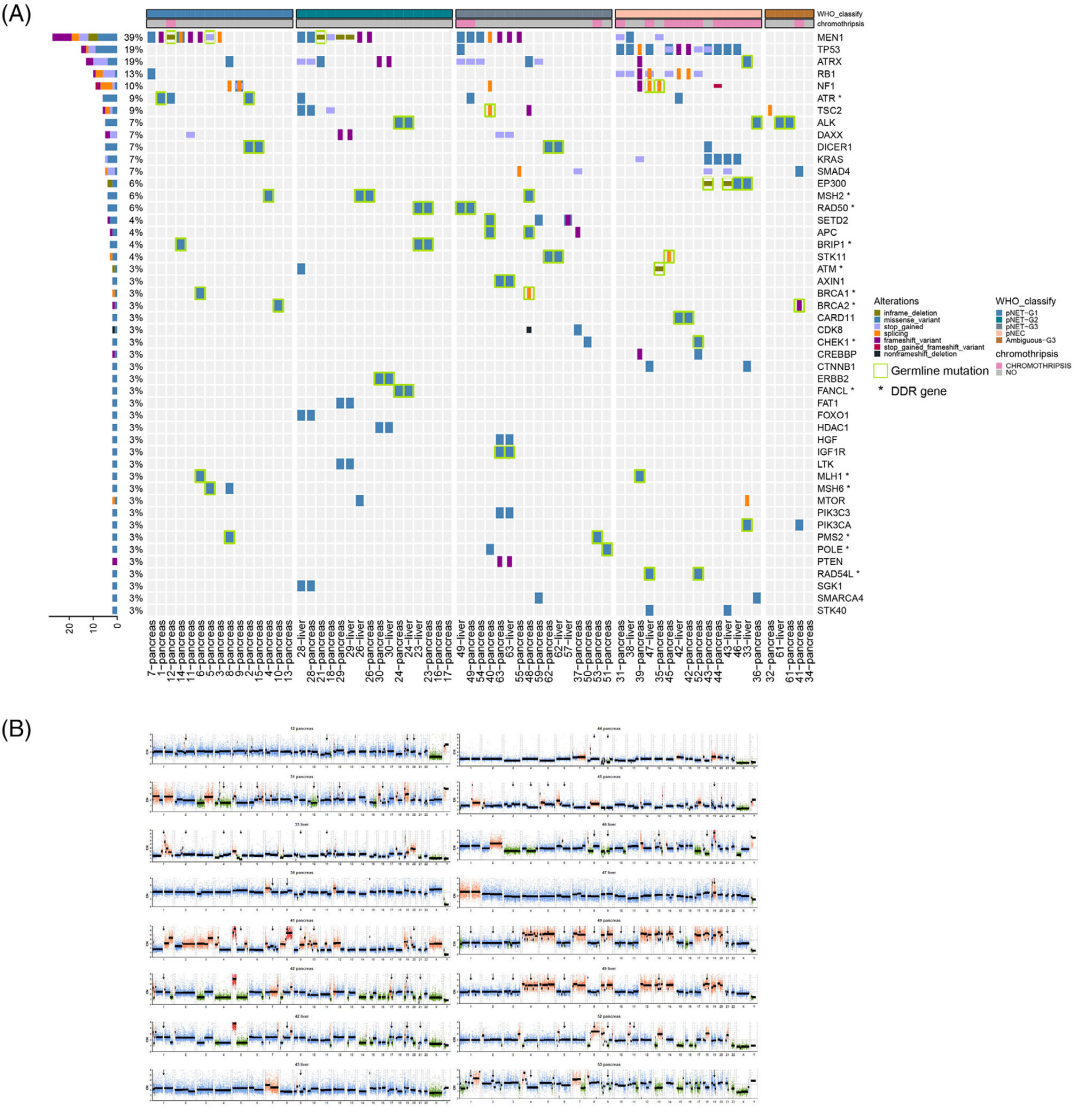
Key gene mutations in 55 patients with pNENs. Various mutation forms are represented by different colors. Germline mutation and DDR gene are marked with a green border and *, respectively (A). Chromothripsis involving different chromosomes in 14 patients. Chromosomes and copy number (CN) are shown on the *x*‐axis and *y*‐axis, respectively. Different CN are represented by different colors (red: CN ≥ 5; orange: 5 > CN ≥ 3; blue: 3 > CN ≥ 1; green: CN < 1). The black arrows indicate the chromosome affected by chromothripsis (B).

The sequencing of 55 patients revealed 245 mutations in 94 different genes. The mutations per case ranged from 1 to 12, with a mean of 4.6. At least one DDR gene alteration was found in 29 patients. *TP53* and *RB1* were affected in 11 and eight patients, respectively. *MEN1* and *ATRX*/*DAXX* were affected in 21 patients and 13 patients, respectively. All patients’ recurrent mutations (3% or higher) are shown in Figure 1A. Other mutations that only occur in single cases were included in Figure S2.

### 2.2 Chromothripsis in patients with pNENs

Chromothripsis has been reported to potentially lead to the rapid malignant progression of some tumors. In our study, we analyzed the occurrence of chromothripsis in pNENs. In total, 14 out of 55 patients had chromothripsis involving different chromosomes. We detected chromothripsis on chromosome 9 in eight cases, chromosome 8 in seven cases, chromosome 1 in six cases, chromosome 4/5/19 in five cases, chromosome 2/6/11 in four cases, chromosome 7/10 in three cases, chromosome 3/13/18/20/21 in two cases, and chromosome 12/14/15/16/17 in one case (Figure 1B). Chromothripsis‐positive patients presented a higher *TP53* alteration (*p* < 0.001) and most of them (10 out of 14) were in the pNECs group (*p* = 0.001). However, there was no significant difference in DDR alteration (*p* = 0.702) and liver metastasis (ENETS Stage IV) at pathological diagnosis (*p* = 0.098) (Table 2).

**TABLE 2:** Comparison of patients with/without chromothripsis.

		Chromothripsis positive	Chromothripsis negative	
		(*N* = 14)	(*N* = 41)	
	Total	No. (%)	No. (%)	*p* Value
Any DDR alterations				0.702
Yes	29	8 (28%)	21 (72%)	
No	26	6 (23%)	20 (77%)	
*TP53* alteration				<0.001
Yes	11	9 (82%)	2 (18%)	
No	44	5 (11%)	39 (89%)	
Diagnosis category				0.001
pNETs	38	3 (8%)	35 (92%)	
pNECs	13	10 (77%)	3 (23%)	
Ambiguous cases	4	1 (25%)	3 (75%)	
ENETS stage^a^				0.098
I/II/III	38	7 (18%)	31 (82%)	
IV	17	7 (41%)	10 (59%)	

^a^ It was evaluated on the day of pathological diagnosis.

### 2.3 Chromothripsis defined a group of patients with poor overall survival

**FIGURE 2:**
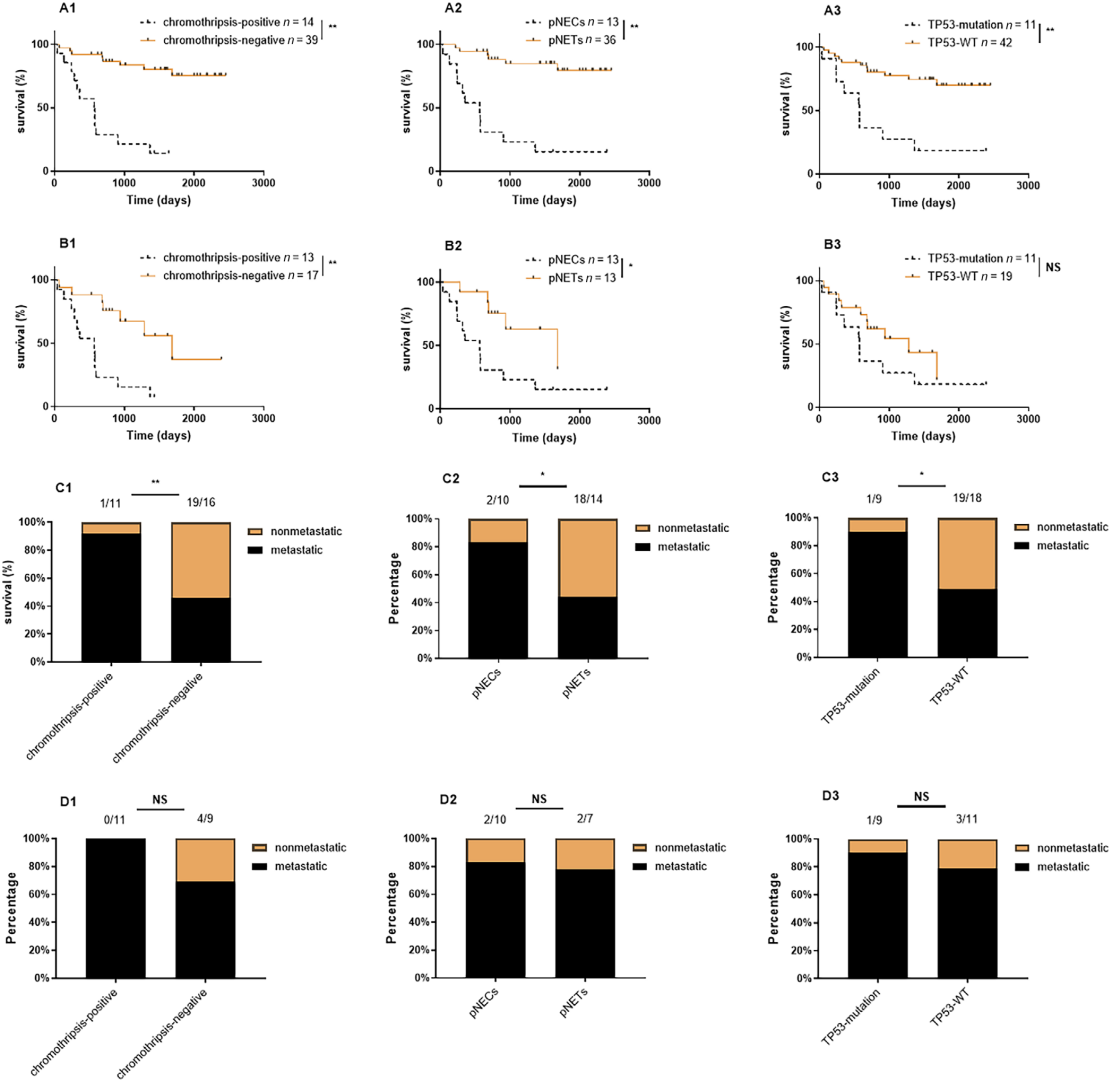
The association between different classifications and OS/distant metastasis (NS for no significance, * for *p* < 0.05, and ** for *p* < 0.01). The Kaplan–Meier curves of different classifications in all pNENs (A1–A3) and in high‐grade pNENs (B1–B3) are shown. The proportion of distant metastasis under different classifications in all pNENs (C1–C3) and in high‐grade pNENs (D1–D3) are shown.

By June 2020, 33 patients had survived, 20 had died, and two had lost follow‐up. In the entire cohort, patients with chromothripsis had a significantly shorter overall survival (OS) of 658 days (95% CI, 389−927) compared with 2056 days (95% CI, 1809−2303) for those without chromothripsis (log‐rank *p* < 0.001). In the high‐grade (G3) pNENs group, patients with chromothripsis also had a shorter OS of 567 days (95% CI, 340−794) compared with 1534 days (95% CI, 1089−1979) for those without chromothripsis (log‐rank *p* = 0.001). The association between different classifications and OS is shown in Figure 2A,B.

In the entire cohort, sex, *TP53* alteration, grade, ENETS Stage IV, and chromothripsis were significantly correlated with OS. The multivariable analysis showed ENETS Stage IV and chromothripsis are independent prognostic indicators for OS. In the high‐grade pNENs group, only grade, ENETS Stage IV, and chromothripsis were significantly correlated with OS. The multivariable analysis showed that only chromothripsis remains an independent prognostic indicator for OS (Table 3).

**TABLE 3:** Univariable and multivariable analyses for OS.

	Patients with high‐grade (G3) pNENs		All patients with pNENs	
	HR (95% CI)	*p*	HR (95% CI)	*p*
Univariable analysis				
Age (≥60y vs. < 60y)	1.268 (0.514–3.125)	0.606	1.760 (0.728–4.254)	0.209
Sex (male vs. female)	2.146 (0.806–5.714)	0.126	2.984 (1.142–7.798)	0.026
DDR alterations (yes vs. no)	0.513 (0.194–1.360)	0.180	0.753 (0.311–1.824)	0.530
*TP53* alteration (yes vs. no)	1.905 (0.769–4.718)	0.164	4.906 (2.003–12.014)	0.001
Histopathology (pNECs vs. pNETs)	3.551 (1.222–10.318)	0.020	9.741 (3.513–27.010)	<0.001
ENETS Stage (IV vs. I/II/III)*	2.804 (1.069–7.354)	0.036	8.505 (3.235–22.363)	<0.001
Chromothripsis (yes vs. no)	4.668 (1.717–12.689)	0.003	8.463 (3.267–21.921)	<0.001
Multivariable analysis				
Sex (male vs. female)	2.775 (0.809–9.518)	0.105	2.786 (0.868–8.943)	0.085
*TP53* alteration (yes vs. no)	0.207 (0.040–1.084)	0.062	0.362 (0.073–1.800)	0.214
Histopathology (pNECs vs. pNETs)	2.468 (0.603–10.097)	0.209	4.263 (0.806–22.535)	0.088
ENETS stage (IV vs. I/II/III)^a^	1.911 (0.542–6.743)	0.314	5.506 (1.742–17.408)	0.004
Chromothripsis (yes vs. no)	5.931 (1.857–18.948)	0.003	4.216 (1.315–13.521)	0.016

^a^ It was evaluated on the day of pathological diagnosis.

Interestingly, two chromothripsis‐negative patients, whose pathological diagnosis was pNECs, had a survival time of 1617 and 2389 days (from the day of the pathological diagnosis to June 2020), respectively. Conversely, one chromothripsis‐positive patient whose pathological diagnosis was pNETs G3 died 280 days after diagnosis. Furthermore, in the ambiguous group (*n* = 4, pNETs G3 or pNECs cannot be clarified by pathologists), one chromothripsis‐positive patient also had shorter OS than three chromothripsis‐negative patients.

### 2.4 Chromothripsis‐positive patients had a higher risk of distant metastasis

By June 2020, 10 patients had distant metastasis after follow‐up. In the entire cohort, patients with chromothripsis had a significantly higher risk of distant metastasis (92%) compared with those without chromothripsis (46%) (*p* = 0.005). In the G3 pNENs group, patients with chromothripsis tended to have a higher risk of distant metastasis (100%) compared with those without chromothripsis (69%) although there was no significant difference (*p* = 0.098) due to the low number of patients in this subset. The association between different classifications and distant metastasis is shown in Figure 2C,D.

### 2.5 *TP53* mutations in chromothripsis‐positive patients preceded chromothripsis

**FIGURE 3:**
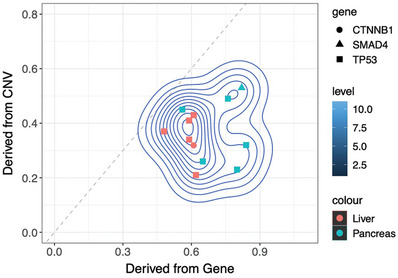
*TP53* mutations in chromothripsis‐positive patients preceded chromothripsis. The chromothripsis‐containing tumor cell fraction was shown in *y*‐axis, and the SNV‐containing tumor cell fraction was shown in *x*‐axis. Points with *y*/*x* < 1 denotes a scenario where SNV mutation occurred before chromothripsis, because the SNV‐containing cell population is larger.

*TP53* mutation has been reported to potentially lead to chromothripsis in cells. Conversely, the occurrence of chromothripsis in cells can impact various key genes, thereby promoting the rapid malignant progression of tumors. To explore such a relationship in pNENs, an algorithm using sequencing data was developed to infer the timing of driver mutation, like *TP53* mutation, relative to chromothripsis events. We calculated the tumor fraction in the same specimen by CNV and driver mutation, respectively. If the tumor fraction calculated by CNV is higher than by driver mutation, it means that the chromothripsis occurred earlier; otherwise, the driver mutation occurred earlier. The results showed that all *TP53* mutations in chromothripsis‐positive patients preceded chromothripsis. *CTNNB1* mutation in patient 33 and *SMAD4* mutation in patient 41 also occurred before chromothripsis (Figure 3).

## 3 DISCUSSION

We conducted a genetic analysis of patients with pNENs at our medical institution. The results showed that in high‐grade pNENs, 10 out of 13 patients whose pathological diagnosis was pNECs and nine out of 11 patients with *TP53* mutation (a characteristic of pNECs) had chromothripsis. By contrast, only two out of 13 patients classified as pNETs G3 and four out of 19 patients without *TP53* mutation had chromothripsis. Subsequent examination revealed that chromothripsis correlates with notably reduced OS and an increased likelihood of distant metastasis. In contrast, neither *TP53* mutations nor pathological pNECs can independently predict the prognosis of G3 pNENs. Thus, we believe that chromothripsis can help differentiate pNECs from high‐grade pNETs and potentially offer prognostic insights and guidance regarding distant metastasis.

The WHO has categorized high‐grade pNENs into pNETs G3 and pNECs to facilitate clinical management and prognosis. However, its heavy reliance on subjective morphological assessment can result in challenges such as inter‐pathologist variability and difficulty distinguishing in certain cases.^14^ In our study, two chromothripsis‐negative patients with the pathological diagnosis of pNECs had long‐term survival. In contrast, one chromothripsis‐positive patient with the pathological diagnosis of pNETs G3 died 280 days after diagnosis. In ambiguous cases, one chromothripsis‐positive patient also exhibited shorter OS compared with three chromothripsis negative. More importantly, the multivariable analysis of high‐grade pNENs showed that only chromothripsis was an independent prognostic indicator for OS. These findings suggested that chromothripsis may be a more reliable predictor for the prognosis of G3 pNENs.

Mechanistically, these results are likely to be related to the influence of chromothripsis on tumor cells. As mentioned before, chromothripsis has the potential to generate massive protumourigenic mutational changes in a relatively short time, which could give tumor cells the advantages of rapid cancer evolution and highly aggressive behavior. As a result, chromothripsis has a more direct and close relationship with rapid progression, and detection of it in high‐grade pNENs may help better identify pNECs, which are usually considered to have a higher malignant degree and a worse prognosis than pNETs G3.

Several reports have shown that the DDR gene and *TP53* can be affected in pNENs.^16^, ^18^, ^34^, ^35^, ^36^ Our results are in line with these studies, however, neither DDR gene nor *TP53* alteration was found to be associated with the survival of pNENs. The specific role of these mutations in the development of pNENs has not been described in these previous studies. But in fact, both DDR gene alteration and *TP53* mutation may be associated with chromothripsis. Alteration in DDR may be a cause of chromothripsis.^37^, ^38^, ^39^, ^40^ In our study, at least one DDR gene alteration was found in 29 patients (53%), and a number of cases were germline mutations suggesting some pNENs had defects in DNA repair at a very early stage, which may provide a premise for the occurrence of chromothripsis. However, in the end, only 14 patients had chromothripsis, indicating other factors may be involved. *TP53* deficiency can facilitate cell survival following DNA damage and may predispose cells to chromothripsis.^41^, ^42^, ^43^, ^44^ Consistent with this, our research also found that chromothripsis‐positive pNENs were more likely to have *TP53* mutation than chromothripsis negative. Then, we noticed that all *TP53* alterations were somatic mutations. It is, therefore, likely that *TP53* mutation may occur after germline mutations in DDR genes. To determine the timeline between *TP53* mutation and chromothripsis, we also did an investigation of nine chromothripsis‐positive patients with *TP53* mutation. Our timing analysis showed that all *TP53* mutations preceded the chromothripsis. Considering the prognosis of pNECs is still dismal under standard platinum combination regimens, we believe further detailed studies for such genetic alterations above may provide important clues for future research searching for related targeted drugs.

In conclusion, we described chromothripsis in high‐grade pNENs in detail for the first time, and it is clearly a catastrophic event defining a consistent group of patients with poor prognosis. Then, we demonstrated that chromothripsis was more linked to aggressive tumors than other known indicators. The main limitation of our study is the low number of patients with G3 pNENs due to its rarity. Larger studies through multi‐center cooperation are required in the future to study the role of chromothripsis in pNENs further.

## 4 METHODS

### 4.1 Patients and pathologic re‐evaluation

It was a retrospective study and approved by the Ethics Committee of West China Hospital, Sichuan University. High‐grade pNENs were searched from patients who underwent biopsy or surgical resections of their tumors (from 2008 to 2018) and had a definite pathological diagnosis of pNENs with mitotic rate > 20/10 HPF or Ki‐67 index > 20%. Cases were excluded if they contained a non‐neuroendocrine component or the pancreas could not be diagnosed as the primary site. Some samples with a definite pathological diagnosis of low‐grade pNETs were also enrolled as a reference to better distinguish high‐grade (G3) pNETs from pNECs. For certainty, all samples included were histologically examined again by our pathologists, and each case was also reassessed for mitotic indices and Ki‐67 labelling index. There were no changes in them relative to the original diagnoses. Then, we graded all pNENs we collected (from 2008 to 2018) according to the newest WHO 2017 classification. The diagnostic review was blinded to molecular genetic findings in our study. Formalin‐fixed and paraffin‐embedded tumor tissues and paired normal tissues were retrieved from the files of the Department of Pathology, West China Hospital, Sichuan University. Clinical information was obtained from related Electronic Case Report Forms.

### 4.2 DNA extraction and targeted next‐generation sequencing

Genomic DNA was extracted from slides of tumor samples and paired normal tissues with MagPure Tissue DNA DF Kit (Magen Inc.; MD5112‐TL‐06) according to the manufacturer's protocol. Extracted DNA was quality‐controlled by Qubit dsDNA HS assay (Thermo Fisher Scientific) and Agilent 2100 Fragment Analyzer. We processed the DNA according to a modified single‐stranded DNA sequencing library preparation protocol. Briefly, genomic DNA was sonicated with 3′‐poly‐A tailed with terminal transferase, ligated with a poly‐dT‐tailed P5 sequencing adaptor, and subjected to several cycles of linear amplification using a P5 sequencing primer. A P7 adaptor with a random nucleotide 3′ overhang was then ligated to the 3′ end of the linear amplification product. From there, the sequencing library was amplified using P7+P5 primers to a sufficient amount. Hybrid capture was performed using standard DNA‐probe‐capture practice. The post‐capture libraries were sequenced on Illumina Novaseq.

For panel sequencing, a customized target capture panel targeting 341 genes (Euler Technology), which covers common oncogenes, tumor suppressor genes, as well as genes of interest for clinical oncology, was used. The panel also included ∼1076 common SNP (common in East Asian population, with MAF in [0.25, 0.8]), which evenly span the genome for copy number variation analysis. The capture assay was validated using external and internal standards to meet a limit‐of‐detection of 0.5% for short nucleotide variation (SNV) at a given standard sequencing depth (∼2000× deduplicated). Genes were identified as DDR‐related in our study based on Pubmed searches^45^, ^46^ and authoritative databases (such as GenCards Database). Specifically, they included *MLH1*, *MSH2*, *MSH6*, *MRE11A*, *PMS1*/*2*, *BRCA1*/*2*, *PARP1*/*2*, *RAD50*, *RAD54L*, *BRIP1*, *BARD1*, *FANCA*, *FANCC*, *FANCL*, *ERCC3*, *ATR*, *ATM*, *CHEK1*/*2*, and *POLE* in this study.

### 4.3 Chromothripsis detection

In our study, chromothripsis was analyzed by pattern recognition aided by machine learning, with a bioinformatic pipeline adapted from ShatterSeek (https://github.com/parklab/ShatterSeek) that modified for capture panel sequencing data. The chromothripsis‐positive threshold was set as “three or more consecutive genomic regions with typical copy number sequence (monotonic and linear change in log copy number) as generated by bridge‐fusion‐bridge‐class, or four or more consecutive genomic regions with typical copy number sequence (repetitive oscillating change in log copy number) as generated by chromosome scattering.” The absolute integer copy number and the exact physical position of each genomic region were computed by linear regression of GC‐adjusted, read‐depth‐normalized, circular segmented output from CNVkit.

### 4.4 Timing of *TP53* mutation and chromothripsis

Chromothripsis is a genome‐wide CNV pattern for which multiple copy number variations across the genome arise from a single incidence of mutation event. We consider a scenario that a cell first gained a mutation (either *TP53* or chromothripsis) to form a colony, and then a second mutation event occur in a subset of these cells. Hence, the tumor population having the first mutation should be larger compared to the population with both mutations. The mutation‐containing cell population (so called “tumor fraction,” for simplicity) was calculated as follow:

1. For *TP53* or any SNV mutant, the SNV‐containing tumor fraction (in percentage) was calculated by Equation (1):
$$\frac{\mathrm{VA}F_{\mathrm{TP}53}*CN_{\mathrm{TP}53-\mathrm{loci}}}{CN_{\mathrm{TP}53-\mathrm{mutant}\;\mathrm{allele}}}$$
  Denoting the variant allelic frequency of *TP53* mutant ($\mathrm{VA}F_{\mathrm{TP}53}$) adjusted by allelic copy ratio of *TP53* ($\frac{CN_{\mathrm{TP}53-\mathrm{loci}}}{CN_{\mathrm{TP}53-\mathrm{mutant}\;\mathrm{allele}}})$ in the tumor cell. For example, a *TP53* mutant allele (heterozygous, 1 copy on a 2‐copy chromosome 17 background) of 25% VAF would translate to a 50% tumor fraction. On the other hand, a *TP53* mutant allele (homozygous, 2 copies, on a 2‐copy chromosome 17 background) of 25% VAF would translate to a 25% tumor fraction. To compute this, the total copy number of the SNV‐containing genomic region as well as the mutant allelic copy number were called by using allelic frequency from common SNP probes on the genomic region with a method similar to B allele frequency analysis in microarray.
2. For chromothripsis, we have the following Equation (2) to calculate the tumor fraction:
$$\vec{y}=\mathrm{tf}\;\times \;\vec{x}+e$$

Where $\vec{y}$ denotes the observed copy number ratio of each CNV segment (in the form of log(2) −1), $\vec{x}$ denotes the real copy number change in tumor cells corresponding to these CNV segments, tf denotes the tumor fraction, and e denotes for regression residual. One shall notice that all $\vec{x}$ are integers, and all $\vec{y}$ are resulted from a gaussian process of observation (by library preparation, targeted capture, sequencing, etc).

To compute the tumor fraction with chromothripsis, we first select choose genomic regions containing the copy number variation related to chromothripsis. Then, we calculate exon‐wise copy number log‐ratio of these genomic regions using CNVkit. For each “integer” of $\vec{x}$, the observed copy number log‐ratios resulted from a probability function, which could be modeled as a gaussian process. Hence, a gaussian mixture model was applied to all exon‐wise copy number log‐ratios to decompose them into several possible gaussian distributions (3), each with a mean log‐ratio change corresponding to an “integer” in $\vec{x}$. Linear regressions were performed with all possible integer combinations of $\vec{x}$ (for example, for a 5‐mode distribution of copy number log‐ratio, the vector of $\vec{x}$ would choose from five integer elements, such as [−1,0,1,2,3], [−2,−1,1,2,4], [1,2,3,4,5], etc.) to $\vec{y}$ according to the abovementioned Equation (2). To make the computation possible, we match an integer to all CNV region belonging to a single mode of gaussian distribution in (3).

A best fit solution was taken at the adjusted *p* < 0.01 and *R* ^2^ > 0.95 for the linear regression solution. By extracting the slope coefficient from this best‐fit linear regression, we obtain the tumor fraction of chromothripsis‐containing tumor cells.

The abovementioned Equations (1) and (2) were validated with pure cancer cell line sequencing data as well as artificial mixtures of cancer DNA into normal PBMC genomic DNA to show that in these cases, both SNV‐inferred and CNV‐inferred tumor fraction were accurate.

### 4.5 Statistical analysis

The OS was assessed as the period from the pathological diagnosis to death or last follow‐up. It was estimated using the Kaplan–Meier method and compared with the log‐rank test. Uni‐ and multivariable Cox regression analyses were used to evaluate the impact of selected clinicopathologic factors. The HRs and 95% CIs were calculated. Differences in characteristics by groups were tested by Chi‐squared or Fisher exact test. Statistical analysis was conducted using SPSS 25.0 software (IBM Corporation, Armonk, New York). A two‐tailed *p* value < 0.05 was considered statistically significant.

## AUTHOR CONTRIBUTIONS

*Conceptualization*: Ming‐Yi Zhang and Ji‐Yan Liu. *Data curation*: Ming‐Yi Zhang, Du He, Yi Zhang, Ke Cheng, Hong‐Shuai Li, Yu‐Wen Zhou, and Qiong‐Xian Long. *Formal analysis*: Ming‐Yi Zhang, Du He, and Ji‐Yan Liu. *Funding acquisition*: Ji‐Yan Liu. *Investigation*: Ming‐Yi Zhang, Du He, Ke Cheng, Hong‐Shuai Li, Yu‐Wen Zhou, and Ji‐Yan Liu. *Methodology*: Ming‐Yi Zhang, Yi Zhang, Ke Cheng, Hong‐Shuai Li, and Yu‐Wen Zhou. *Project administration*: Ji‐Yan Liu. *Resources*: Ming‐Yi Zhang, Du He, Yi Zhang, Ke Cheng, Hong‐Shuai Li, Qiong‐Xian Long, Yu‐Wen Zhou, and Ji‐Yan Liu. *Software*: Ming‐Yi Zhang, Yi Zhang, Ke Cheng, and Yu‐Wen Zhou. *Supervision*: Ji‐Yan Liu. *Validation*: Ming‐Yi Zhang, Du He, Yi Zhang, and Rui‐Zhi Liu. *Visualization*: Ming‐Yi Zhang, Yi Zhang, Ke Cheng, and Hong‐Shuai Li. *Writing‐original draft*: Ming‐Yi Zhang and Yi Zhang. *Writing—review and editing*: Ming‐Yi Zhang, Yi Zhang, Rui‐Zhi Liu, and Ji‐Yan Liu. All authors have read and agreed to the published version of the manuscript.

## CONFLICT OF INTEREST STATEMENT

The authors report no conflicts of interest.

## ETHICS STATEMENT

All procedures followed were under the ethical standards of the committee responsible for human experimentation (institutional and national) and with the Helsinki Declaration of 1964 and later versions. This was a retrospective study and approved by the Ethics Committee of West China Hospital, Sichuan University (ethics approval number 240597).

## Supporting information

Supporting information

## Data Availability

Data are available from the corresponding author (Ji‐Yan Liu: liujiyan1972@163.com) by reasonable request.
