# Supplementary material for: Chromothripsis is a novel biomarker for prognosis and differentiation diagnosis of pancreatic neuroendocrine neoplasms
Source: MedComm (2020). 2024 Jul 10;5(7):e623. doi: 10.1002/mco2.623 (PMC11234462; doi:10.1002/mco2.623)
Supplement: Supplementary file 1 — Supporting information [file MCO2-5-e623-s001.docx]

**Chromothripsis is a Novel Biomarker for Prognosis and Differentiation Diagnosis of Pancreatic Neuroendocrine Neoplasms**


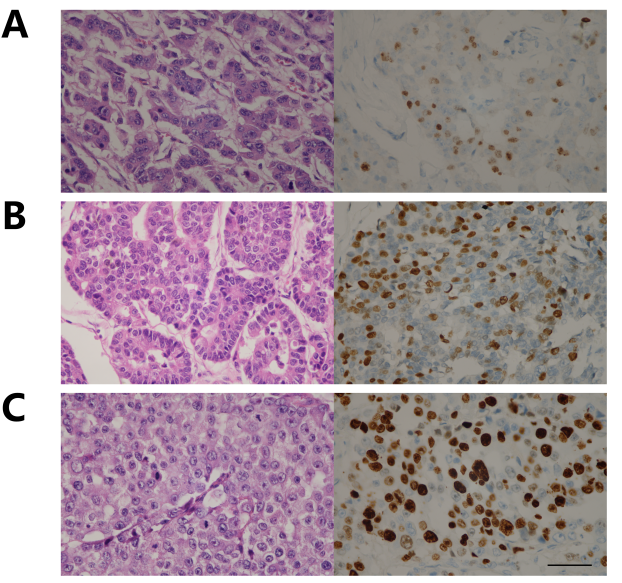


**Supplemental Figure 1.** Representative histopathological images and corresponding Ki-67 immunohistochemistry results (magnification 400×) for pNETs G3 (A), ambiguous cases (B), and pNECs (C). The scale bar represents 50 μm.


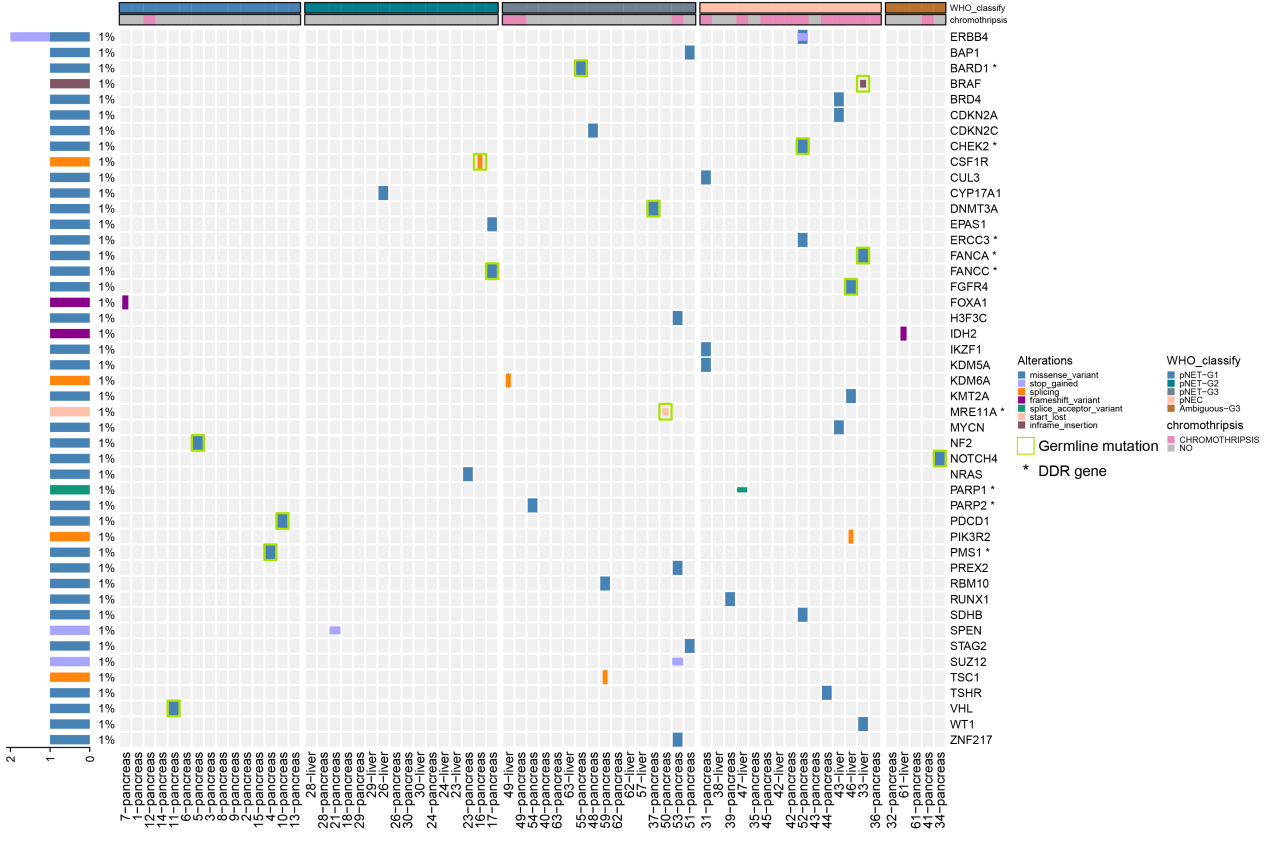


**Supplemental** **Figure 2.** Gene mutations that only occur in single cases in 55 pNENs patients. Different colours represent various mutation forms. Germline mutation and DDR gene are marked with a green border and *, respectively.

**Supplementary Table 1.** Pathologic and Genetic Features of each G3 Patient

| **Case** | **Specimen source** | **Diagnosis** | **Ki-67 (%)** | **Mitotic rate** | ***TP53* mutation** | **Chromothripsis** | **OS** |
| --- | --- | --- | --- | --- | --- | --- | --- |
| 31 | Pancreas | NEC | 80 | 28 | + | + | 566 |
| 32 | Pancreas | Ambiguous | 50 | 23 | - | - | 64 |
| 33 | Liver | NEC | 80 | 64 | - | + | 132 |
| 34 | Pancreas | Ambiguous | 22 | 10 | - | - | 1282 |
| 35 | Pancreas | NEC | 80 | 30 | - | - | 1617 |
| 36 | Pancreas | NEC | 30 | 30 | - | + | 319 |
| 37 | Pancreas | NET | 50 | 10 | - | - | 940 |
| 38 | Liver | NEC | 60 | 65 | + | - | 244 |
| 39 | Pancreas | NEC | 50 | 11 | + | - | 2389 |
| 40 | Pancreas | NET | 30 | 10 | - | - | 1683 |
| 41 | Pancreas | Ambiguous | 40 | 15 | - | + | 592 |
| 42 | Pancreas | NEC | 60 | 40 | + | + | 239 |
| 42 | Liver | NEC | 60 | 38 | + | + | 239 |
| 43 | Pancreas | NEC | 40 | 20 | + | - | 575 |
| 43 | Liver | NEC | 50 | 36 | + | + | 575 |
| 44 | Pancreas | NEC | 50 | 32 | + | + | 355 |
| 45 | Pancreas | NEC | 50* | 18* | + | + | 576 |
| 46 | Liver | NEC | 60 | 28 | + | + | 907 |
| 47 | Liver | NEC | 60 | 14 | + | + | 1364 |
| 48 | Pancreas | NET | 30 | 16 | - | - | 690 |
| 49 | Pancreas | NET | 10 | 1 | - | + | 1428 |
| 49 | Liver | NET | 40 | 40 | + | + | 1428 |
| 50 | Pancreas | NET | 30 | 10 | - | - | 1688 |
| 51 | Pancreas | NET | 30 | 10 | - | - | 824 |
| 52 | Pancreas | NEC | 80 | 150 | + | + | 35 |
| 53 | Pancreas | NET | 40 | 15 | - | + | 280 |
| 54 | Pancreas | NET | 40 | 25 | - | - | 525 |
| 55 | Pancreas | NET | 25 | 21 | - | - | 1009 |
| 57 | Liver | NET | 22 | 29 | - | - | 682 |
| 59 | Pancreas | NET | 25 | 15 | - | - | 1437 |
| 61 | Pancreas | Ambiguous | 22 | 14 | - | - | 1018 |
| 61 | Liver | Ambiguous | 30* | 12* | - | - | 1018 |
| 62 | Pancreas | NET | 25 | 17 | - | - | 784 |
| 62 | Liver | NET | 25 | 18 | - | - | 784 |
| 63 | Pancreas | NET | 25 | 13 | - | - | 737 |
| 63 | Liver | NET | 15* | 1* | - | - | 737 |

*****The specimen was obtained through biopsy.
